# Supplementary material for: A Likelihood Approach for Real-Time Calibration of Stochastic Compartmental Epidemic Models
Source: PLoS Comput Biol. 2017 Jan 17;13(1):e1005257. doi: 10.1371/journal.pcbi.1005257 (PMC5240920; doi:10.1371/journal.pcbi.1005257)
Supplement: S1 File — (TAR.GZ) [file pcbi.1005257.s014.tar.gz › HSPH_Online-SI-Revision/PF08/n1-mild/n1-mild_PF2_summary2.pdf]

peak −8	3w spec	3w cum	5w spec	5w cum	end	3w spec LOG	3w cum LOG	5w spec LOG	5w cum LOG	end LOG
mean	443.	362.4	191.	272.4	58.7	0.6	0.6	0.5	0.5	0.3
median	226.9	231.7	128.2	186.4	53.4	0.6	0.6	0.5	0.5	0.3
quantile .1	80.2	102.9	61.1	97.5	43.2	0.3	0.4	0.3	0.3	0.2
quantile .9	843.	590.5	340.8	489.7	73.8	0.9	0.8	0.7	0.3	0.5
44	40	48	40	50						

peak −4	3w spec	3w cum	5w spec	5w cum	end	3w spec LOG	3w cum LOG	5w spec LOG	5w cum LOG	end LOG
mean	109.6	122.6	88.8	86.3	56.8	0.4	0.4	0.5	0.4	0.3
median	71.6	85.6	60.9	61.3	54.3	0.4	0.4	0.5	0.4	0.3
quantile .1	49.2	51.3	50.1	47.7	40.2	0.3	0.2	0.3	0.2	0.2
quantile .9	184.4	222.4	179.6	157.2	76.1	0.6	0.5	0.6	0.2	0.4
46	43	44	44	49						

peak	3w spec	3w cum	5w spec	5w cum	end	3w spec LOG	3w cum LOG	5w spec LOG	5w cum LOG	end LOG
mean	83.3	71.1	523.4	70.9	72.6	0.5	0.4	0.6	0.4	0.4
median	68.	58.6	75.	60.7	61.2	0.5	0.4	0.5	0.4	0.4
quantile .1	49.8	46.6	56.3	44.2	48.6	0.3	0.2	0.3	0.3	0.3
quantile .9	127.4	118.9	144.	109.3	103.3	0.6	0.5	0.7	0.3	0.6
49	49	48	49	48						

peak +4	3w spec	3w cum	5w spec	5w cum	end	3w spec LOG	3w cum LOG	5w spec LOG	5w cum LOG	end LOG
mean	391.8	1037.4	1021.7	1471.9	2500.3	0.6	0.5	0.7	0.5	0.6
median	75.6	71.2	107.4	74.5	82.7	0.5	0.4	0.7	0.5	0.6
quantile .1	56.5	49.8	59.1	51.7	56.3	0.3	0.3	0.5	0.3	0.4
quantile .9	299.3	121.7	873.	162.3	297.6	0.8	0.6	1.	0.3	0.9
46	47	48	47	43						

peak +8	3w spec	3w cum	5w spec	5w cum	end	3w spec LOG	3w cum LOG	5w spec LOG	5w cum LOG	end LOG
mean	1277.6	1867.7	1491.1	2703.7	5264.6	0.8	0.7	0.9	0.7	0.9
median	96.8	75.5	100.6	83.6	116.7	0.7	0.6	0.8	0.6	0.8
quantile .1	63.1	60.1	65.3	59.5	63.4	0.5	0.4	0.6	0.4	0.6
quantile .9	5158.	562.	4568.	832.3	1612.7	1.3	0.8	1.2	0.4	1.4
46	46	45	47	39	{}	{}	{}	{}	{}	{}
